# Development of a High-Accuracy, Low-Cost, and Portable Fluorometer with Smartphone Application for the Detection of Urinary Albumin towards the Early Screening of Chronic Kidney and Renal Diseases

**DOI:** 10.3390/bios13090876

**Published:** 2023-09-08

**Authors:** Visarute Pinrod, Wireeya Chawjiraphan, Khoonsake Segkhoonthod, Kriangkai Hanchaisri, Phornpol Tantiwathanapong, Preedee Pinpradup, Thitirat Putnin, Dechnarong Pimalai, Kiatnida Treerattrakoon, Ubon Cha’on, Sirirat Anutrakulchai, Deanpen Japrung

**Affiliations:** 1 National Nanotechnology Center (NANOTEC), National Science and Technology Development Agency (NSTDA), Thailand Science Park, Pathumthani 12120, Thailand; visarute.pin@nanotec.or.th (V.P.); wireeya.cha@nanotec.or.th (W.C.); khoonsake@ayinnova.com (K.S.); kriangkai.hu@gmail.com (K.H.); pinpradup.pd@gmail.com (P.P.); thitirat.put@nanotec.or.th (T.P.); dechnarong.pim@nanotec.or.th (D.P.); kiatnida@nanotec.or.th (K.T.); 2 Department of Pure and Applied Chemistry, Technology and Innovation Centre, University of Strathclyde, Glasgow G1 1RD, UK; 3 Department of Biochemistry, Faculty of Medicine, Khon Kaen University, Khon Kaen 40002, Thailand; ubocha@kku.ac.th; 4 Chronic Kidney Disease Prevention in the Northeast of Thailand (CKDNET), Khon Kaen University, Khon Kaen 40002, Thailand; sirirt_a@kku.ac.th; 5 Department of Internal Medicine, Faculty of Medicine, Khon Kaen University, Khon Kaen 40002, Thailand

**Keywords:** albuminuria, aptasensor, fluorescence quenching, portable fluorometer, chronic kidney disease, renal disease

## Abstract

This study presents the development of a portable fluorometer with a smartphone application designed to facilitate the early screening of chronic kidney and renal diseases by enabling the sensitive detection of urinary albumin. Utilizing a fluorescence-based aptasensor, the device achieved a linear calibration curve (0.001–1.5 mg/mL) with a linearity of up to 0.98022 and a detection limit of 0.203 µg/mL for human serum albumin (HSA). The analysis of 130 urine samples demonstrated comparable performance between this study’s fluorometer, a commercial fluorometer, and the standard automated method. These findings validate the feasibility of the portable fluorometer and aptasensor combination as a reliable instrument for the sensitive and specific measurement of HSA in urine samples. Moreover, the fluorometer’s portability offers potential applications in portable point-of-care testing, enhancing its utility in clinical settings for early disease screening.

## 1. Introduction

Human serum albumin (HSA), the most abundant protein in human plasma, plays an important role in maintaining oncotic blood pressure and transporting various biomolecules through the blood circulatory system. HSA, synthesized exclusively in the liver, contains 585 amino acid residues and has a molecular weight of 60 kDa [1,2,3,4]. Abnormal excretion levels of HSA in the urine (albuminuria) serve as an early marker for screening and monitoring kidney malfunction, often associated with non-communicable diseases such as diabetes mellitus, cardiovascular diseases, glomerulitis, hypertension, and early-stage kidney damage [5,6,7,8,9,10].

In hospitals, traditional methods for detecting albumin in urine primarily rely on immunoassay techniques, including immunoturbidimetric assay, immunonephelometric assay, chemiluminescence immunoassay, radioimmunoassay, fluorescent immunoassay, immunoelectrophoresis, and enzyme-linked immunosorbent assay [11,12,13,14,15]. Although these approaches are highly sensitive and accurate, they are also expensive because they require costly instrumentation. To address this issue, urine dipsticks have been used for albuminuria screening due to their low cost and ease of use [16,17]. However, this method exhibits low sensitivity and provides only semiquantitative results.

Biosensor methods have gained significant popularity as analytical devices in various fields due to their fast response, low cost, and high sensitivity and specificity. Several biological sensing techniques have been explored as alternative tools for detecting albumin in urine and blood samples [18,19,20,21]. The need for a highly selective, rapid, simple, and cost-effective biosensing platform has spurred interest in nanomaterials with unique optical, electronic, and catalytic properties that make them ideal candidates for advanced biosensing systems [22,23]. Graphene, a two-dimensional single-layer carbon material, has garnered attention for its distinctive features such as good water dispersibility, remarkable mechanical strength, and excellent electrical and thermal properties. It has been widely employed as a carbon nanomaterial for developing high-performance sensors capable of detecting various biomolecules, including DNA, miRNAs, proteins, metal ions, and small molecules [24,25,26,27].

In our recent work, we developed a fluorescence-based aptasensor platform using graphene oxide (GO) as a fluorescence-quenching aptasensor [28,29] for the quantitative detection of albumin in urine samples. However, a conventional commercial fluorometer consists of high-cost and large-sized optical components such as xenon lamps and photomultiplier tubes [30]. This results in bulky and expensive fluorometers that are not affordable for healthcare systems in remote areas. Furthermore, commercial handheld fluorometers require the interpretation of measured fluorescence intensities and concentrations [31,32]. While several groups have developed low-cost fluorometer prototypes, these prototypes are still in the early stages and are not specifically designed for albuminuria quantification [33,34,35,36,37]. 

**Figure 1:**
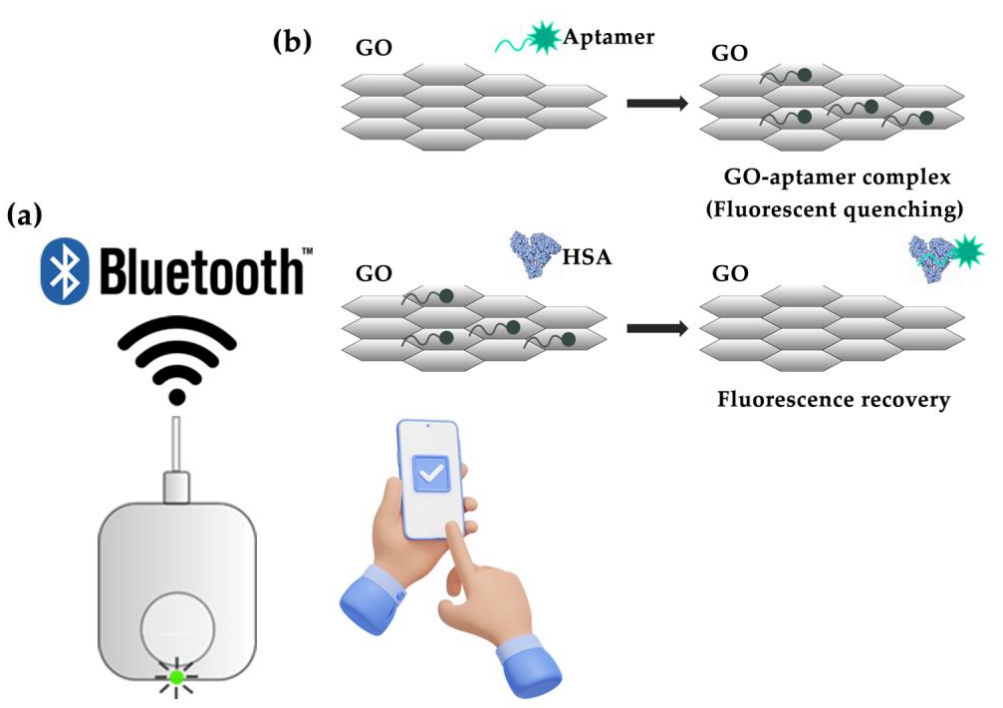
Schematic of the complete device used in this study is presented. The device comprises a fluorometer with an integrated graphene oxide (GO)–aptamer biosensor and a custom smartphone application. (**a**) The fluorometer is combined with the smartphone application. (**b**) An illustration of the GO–aptamer assay principle demonstrates that the fluorescence signal is absent when fluorescence-labeled aptamers bind to GO, resulting in fluorescence quenching. In the presence of the target molecules, the aptamers bind to them, dissociating from GO and leading to the recovery of the fluorescence signal.

In this study, we developed a portable fluorometer and a smartphone application (Figure 1) that allowed an aptasensor to determine HSA in urine samples and quantify albuminuria, potentially aiding in the screening of kidney function abnormalities. 

The aptasensor employs aptamer-labeled fluorescence, a single-stranded DNA designed to target albumin in conjunction with GO, which exhibits fluorescence-quenching properties [28]. The proposed mechanism involves the attachment of the fluorescence-labeled aptamer to specific sites on human serum albumin situated on graphene oxide. This attachment creates an aptamer–graphene complex that leads to fluorescence quenching. Upon the introduction of a sample containing human serum albumin, the aptamer disassociates from the graphene oxide, binds to the albumin, and restores the fluorescence signal. This recovered fluorescence signal corresponds to the concentration of the human serum albumin target in the sample. The portable fluorometer was designed to optimize the performance of the aptasensor, featuring a narrow wavelength spacing of 15 nm between the excitation peak (650 nm) and emission peak (665 nm). These long excitation wavelengths and narrow spacings effectively mitigate undesirable signals from autofluorescence in biological materials found in urine [38]. To ensure accuracy while maintaining affordability, we incorporated small (5 × 5 mm^2^), high-quality (OD 4) optical filters. The fluorometer was designed for mass production, utilizing an injection-molding technique for the case. The fluorometer circuit board was designed for automatic assembly by pick-and-place machines. We combined the portable fluorometer with a smartphone application to calculate, report, and store albumin concentration data. To evaluate the performance of our developed device, we compared its albumin measurement results with those obtained from an immunoturbidimetric assay conducted at a hospital laboratory using samples from 130 volunteers.

## 2. Materials and Methods

### 2.1. Aptamer and Reagents’ Preparation, Aptasensors

In this study, the 87-base single-stranded DNA sequence of the albumin-binding aptamer (5′/Cy5/ATA CCA GCT TAT TCA ATT CCC CCG GCT TTG GTT TAG AGG TAG TTG CTC ATT ACT TGT ACG CTC CGG ATG AGA TAG TAA GTG CAA TCT/3′) or H8 [28,37] was purchased from Integrated DNA Technologies (Singapore). Purified human serum albumin (HSA) was obtained from Sigma-Aldrich (A9731; St. Louis, MO, USA). Lyophilized HSA was dissolved in sterile phosphate-buffered saline (PBS) to prepare a 100 mg/mL stock solution. A calibration curve was generated using different concentrations of HSA to determine the linearity range of the measurements. The HSA standard was prepared by diluting the HSA stock solution with PBS (pH 7.4), resulting in final concentrations ranging from 0.001 to 1.5 mg/mL. Monolayer powder graphene oxide (GO) was synthesized using a modified Hummers’ method and dissolved in sterile ultrapure water to prepare 5 mg/mL stock solutions, as described in our previous study [28]. The solution was stored at 25 °C and used the following day.

### 2.2. Urine Sample Collection

Random spot urine samples were collected from 130 volunteers residing in the Ubonrat district area of Khon Kaen, Thailand, between March and April 2021. Urine samples were collected in sterile screw-cap tubes and used on the same day without any additional pretreatment. Each urine sample was divided into two parts. The first part was analyzed using commercial and developed fluorometers, while the second part was sent to a hospital laboratory to determine the albumin concentration using reference methods. All clinical samples were collected and studied under the ethical approval (Approval number HE601035) granted by the Office of the Khon Kaen University Ethics Committee in Human Research (Institutional Review Board number IRB00001189), Khon Kaen University, Thailand.

### 2.3. Comparison of Urinary Albumin Analysis: Commercial Portable Fluorometer, Developed Fluorometer, and Standard Hospital Method

**Figure 2:**
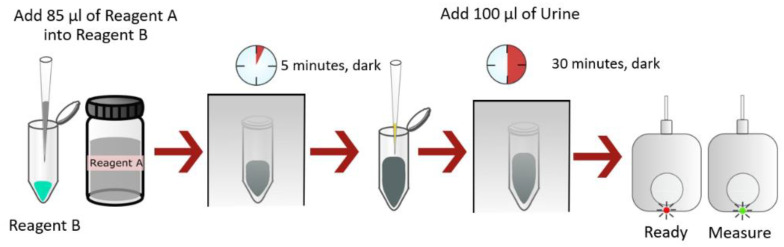
Albumin measurement using our developed aptasensor platform. Reagent A refers to the graphene oxide solution, and reagent B corresponds to fluorescence-labeled aptamer-bound human serum albumin.

Albumin measurements were conducted using a modified GO–aptamer assay, as described in our previous study [28]. Briefly, 15 μL of GO (5 mg/mL) was suspended in 70 μL of PBS to prepare a GO–PBS solution. To form GO–aptamer complexes, 15 μL of 5 μM fluorescence-labeled aptamer (H8) was incubated with 85 μL of the GO–PBS solution for 5 min at room temperature in the dark. Subsequently, 100 μL of the standard albumin solution with concentrations ranging from 0.001 to 1.5 mg/mL (stock concentration) was added to the complex mixture and incubated at room temperature for 30 min to facilitate aptamer–albumin interactions. To detect albumin in the urine samples, undiluted urine was incubated with the GO–aptamer mixture, following the same procedure. The albumin measurements performed using a commercial portable fluorometer and our developed platform are demonstrated in the VDO Supplementary Materials and illustrated in Figure 2, respectively.

The fluorescence signal was measured using a commercial portable fluorometer (Quantus, Promega, Madison, WI, USA) and our developed portable fluorometer at an excitation wavelength of 630 nm and an emission wavelength of 670 nm. To calculate the albumin concentration in the urine samples, a calibration curve of albumin concentration was plotted against the fluorescence intensity. The percentage of the fluorescence response (F) was calculated using Equation (1):

F = 100(Fob − Fmin)/(Fmax − Fmin)(1)

Here, Fmax and Fmin represent the maximal and minimal fluorescence intensities of the fluorescently labeled aptamer with and without graphene (complex), respectively, and Fob represents the fluorescence intensity of the fluorescently labeled aptamer-bound HSA.

HSA concentrations in 130 urine samples were analyzed using our modified aptasensor [28,29], and fluorescence intensities were measured using both a commercial portable fluorometer and our developed device. The results were compared with the concentrations obtained from the turbidimetric immunoassay, which is the standard method used in hospitals (COBAS INTEGRA 400; Roach Diagnostics, Singapore) for albuminuria quantification. Correlation plots were constructed to determine the performances of the developed aptasensor and fluorometer devices.

The data were analyzed using Origin software (version 6.0) and SPSS software (version 20.0) to calculate the Pearson correlation coefficients (r) and *p*-values, respectively. If the *p*-values were less than 0.01, the datasets were considered statistically correlated. Conversely, if the *p*-values were ≥0.01, the datasets were not considered correlated.

### 2.4. High-Accuracy, Low-Cost, and Portable Fluorometer Development

**Figure 3:**
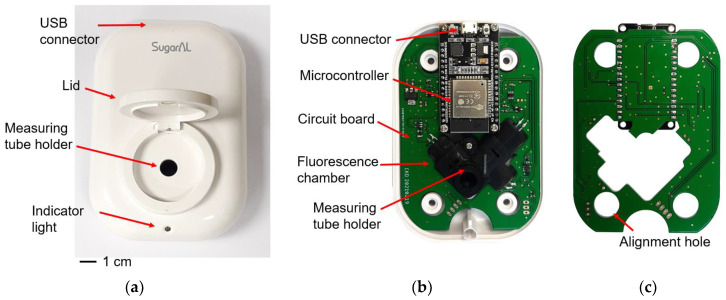
Photographs illustrating the developed portable fluorometer. (**a**) The complete body of the portable fluorometer. (**b**) The front side, showing the connection between the circuit board and the fluorescence chamber. (**c**) The back side of the circuit board, which does not include any devices to support one-sided pick-and-place assembly.

A high-accuracy, low-cost, and portable fluorometer (Figure 3a) was designed to measure the fluorescence intensity of the GO-mediated fluorescence-quenching aptasensor. The fluorometer has a compact size of 11.0 × 7.5 × 4.0 cm^3^, allowing it to be easily held and operated with one hand. The fluorometer lid is opaque, minimizing interference from external light. The case material is acrylonitrile butadiene styrene (ABS), a suitable material for medical applications. ABS possesses a high tensile strength of 29.6–48 MPa, resists chemical reactions, and withstands temperatures of 68–100 °C.

Inside the fluorometer (Figure 3b), a circuit board integrates electronic components with a 3D-printed fluorescence chamber that houses the optical components and provides space for holding the measuring tube. The circuit board primarily utilizes surface-mount devices (SMDs) on the top side, enabling mass production through surface-mount technology component placement systems, also known as pick-and-place machines. The bottom side minimizes the use of SMD components and employs through-hole devices sparingly (Figure 3c). 

#### 2.4.1. Mechanical and Optical Design

**Figure 4:**
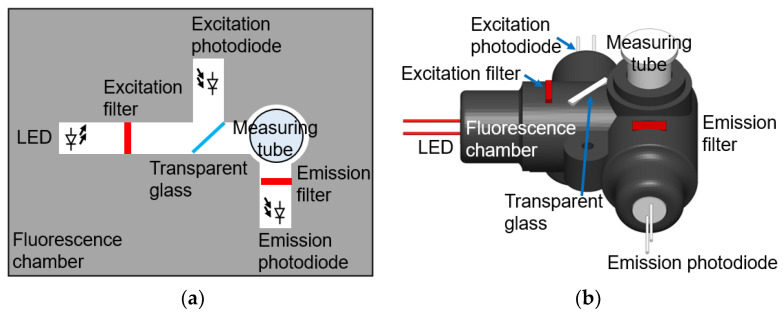
The fluorescence chamber integrates optical components and contains a measuring tube for measuring fluorescence intensity. (**a**) A top view diagram illustrating the fluorescence chamber. (**b**) A 3D model showcasing the integration of the fluorescence chamber with optical components.

The mechanical and optical design involves the integration of optical components within the fluorescence chamber, as depicted in Figure 4, which provides space to accommodate the measuring tube. The optical components, illustrated in Figure 4a, include an LED, an excitation filter, a transparent glass sheet, an excitation photodiode, an emission filter, and an emission photodiode. These components, along with the measuring tube, are inserted into the black opaque fluorescence chamber, as shown in the 3D model presented in Figure 4b. The fluorescence chamber is fabricated using a fused filament fabrication 3D printing technique, enabling rapid adjustments to the design of different measuring tubes and optical components for various applications. To minimize reading errors caused by light reflection and penetration, an opaque matte black polylactic acid filament is utilized.

The employed LED (VLCS5830, Vishay Intertechnology, Malvern, PA, USA) has a central wavelength of 631 nm, aligning with the absorption peak of the Cy5-fluorescence-labeled aptamer. It offers a high intensity of 65,000 mcd and a small angle of half intensity of ±4° to maximize excitation. High-quality excitation and emission filters (Shenzhen Kaitao Optical Technology, Shenzhen, China) with an optical density of 4 were employed for accurate fluorescence intensity measurements. The cost of optical filters was minimized by using small-area filters measuring 5 mm × 5 mm. To select the appropriate excitation wavelength, an excitation bandpass filter with a central wavelength of 625 nm and a full width at half maximum (FWHM) of 30 nm was utilized, considering the partial overlap between the LED spectrum and the emission spectrum. LEDs are preferred over laser diodes due to their longer lifetimes and lower costs [39,40]. Additionally, a transparent glass sheet was placed at a 45° angle to split a portion of the excitation beam toward the excitation photodiode (Osram SFH 213, Osram, Munich, Germany), which measures the excitation beam intensity. For measuring fluorescence intensity, a clear measuring tube was used to contain the sample mixture. The fluorescence light was filtered using an emission bandpass filter with a central wavelength of 668 nm and an FWHM of 15 nm. The emitted fluorescence light intensity was measured using an emission photodiode (Osram SFH 213 PIN photodiode). PIN photodiodes were chosen because they demonstrate acceptable performance and are more cost-effective than avalanche photodiodes and microphotomultiplier tubes.

The case was manufactured using an injection-molding technique to reduce costs during high-volume production. It was designed to be user-friendly and easy to operate. For instance, the symmetric design and small size enable the one-handed operation of the fluorometer. The case thickness ranges from 0.5 to 1.0 mm, ensuring rigidity while keeping the device lightweight.

#### 2.4.2. Circuit Design and Analysis

**Figure 5:**
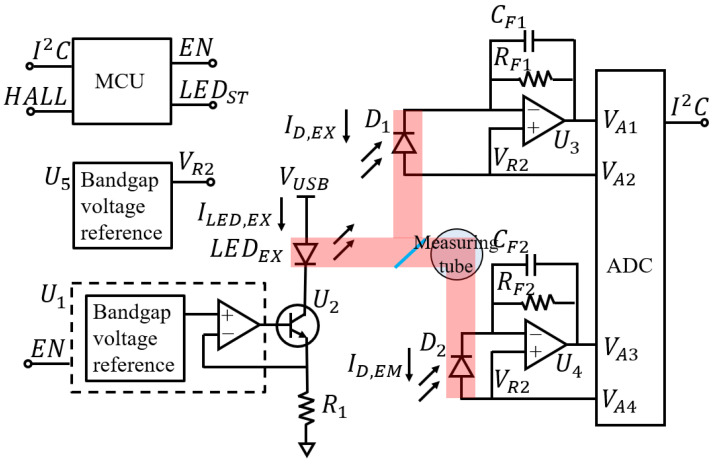
Simplified circuit diagram of the fluorometer.

The circuit board of the fluorometer was designed to accurately quantify albuminuria using low-cost electronic components. Figure 5 illustrates the simplified circuit diagram, which includes a microcontroller, power management, excitation LED driver, excitation photodiode amplifier, and emission photodiode amplifier. The excitation LED driver utilizes a current source circuit with a stable bandgap voltage reference ($U_{1}$, MAX6070B, 2 ppm/°C) and a stable current-sensing resistor ($R_{1}$, MCR03EZPD24R0, 100 ppm/°C). The current flowing through the excitation LED ($I_{LED,EX}$) is calculated as follows:

(2) $$I_{LED,EX}=V_{U1}/R_{1}$$

where $V_{U1}$ is the reference voltage and $R_{1}$ is the resistance of the current sensing resistor.

The excitation light is partially reflected off a transparent glass sheet towards the excitation photodiode ($D_{1}$) to measure the excitation intensity. The fluorescence emitted from the measuring tube is detected using an emission photodiode ($D_{2}$). To minimize errors caused by the photodiode dark current [41], transimpedance amplifiers are employed for both the excitation and emission photodiodes instead of using a photovoltaic mode or shunt resistor. The voltage output is calculated as follows:

(3) $$V_{A1}=V_{R2}+I_{D,EX}R_{F1},$$

Here, $V_{R2}$ represents the reference voltage, $I_{D,EX}$ is the excitation photodiode current, and $R_{F1}$ denotes the resistance of the feedback resistor. To enhance stability and minimize noise and power line interference, the capacitor $C_{F1}$ is incorporated to reduce the bandwidth of the transimpedance amplifier ($f_{EX}=1/2\pi R_{F1}C_{F1}$). Similarly, the voltage output for the emission photodiode reader ($V_{A3}$) is determined as follows:

(4) $$V_{A3}=V_{R2}+I_{D,EM}R_{F2},$$

Here, $I_{D,EM}$ represents the emission photodiode current and $R_{F2}$ denotes the resistance of the feedback resistor. The bandwidth of the emission photodiode amplifier is $f_{EM}=1/2\pi R_{F2}C_{F2}$. The voltage outputs from the photodiode amplifiers are measured using an analog-to-digital converter (ADC, MCP3462RT). A microcontroller (ESP32) facilitates communication with smartphones via Bluetooth, controls the LED, and reads data from the ADC and a Hall sensor for lid-closing detection.

#### 2.4.3. Fluorometer and Smartphone Software

**Figure 6:**
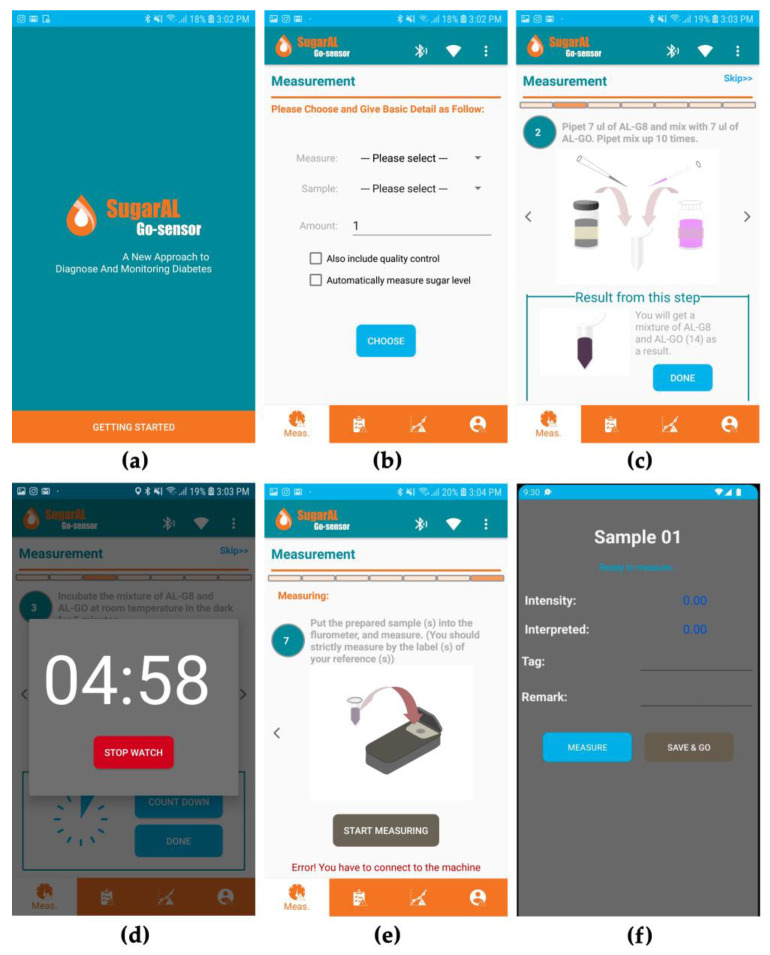
The mobile application used with the fluorometer. (**a**) The software’s welcome screen. (**b**) Options for the user to choose between glycated albumin and HSA. (**c**) An example of instructions guiding users to properly prepare a sample. (**d**) An example of the incubation process during the preparation of a sample. (**e**) The last step of sample preparation, with an error showing that the application is not connected to the machine. (**f**) An example screen when the application receives intensity data from the machine.

A user-friendly smartphone application (Figure 6) was developed using Flutter 2.10.5, an open-source, cross-platform UI software development kit. Users can easily read and track albumin concentrations using iOS and Android phones. The application commands the microcontroller to measure the fluorescence intensity and sends the results to the smartphone via Bluetooth. The albumin concentration is calculated from the fluorescence intensity using a calibration curve. Data are saved in NoSQL and the Firestore database, a real-time document database platform. Users have the option to log into the application to record and track albumin concentrations over an extended period.

To measure albumin concentration, users install the smartphone application, connect the fluorometer to the application via Bluetooth, and follow the instructions on the application until they reach the measurement screens. Users collect and mix urine with solutions prepared according to the protocol explained in Section 2.3. After mixing, the mixture is placed in a measuring tube, and the tube is inserted into the fluorometer. Once the fluorometer lid is closed, the fluorescence intensity is automatically measured and sent to the smartphone. The albumin concentration is calculated, stored, and displayed in the smartphone application.

**Figure 7:**
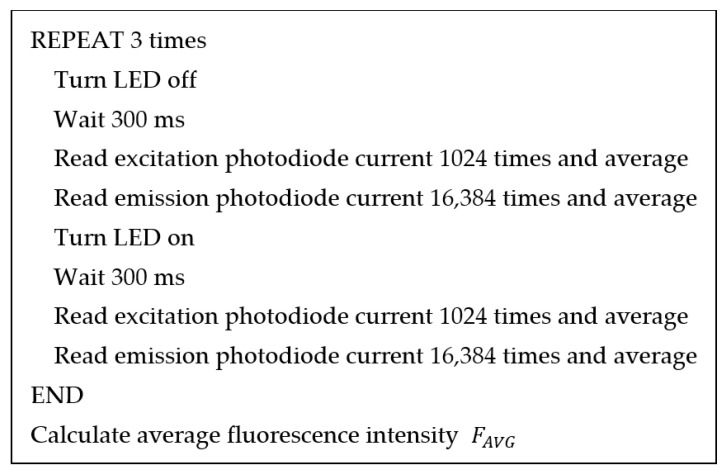
Pseudocode explaining the steps for fluorescence intensity measurement.

Fluorescence intensity measurement is controlled by a microcontroller, as described in the pseudocode in Figure 7. The microcontroller turns off the LED by setting the enable pin (EN) of the voltage reference ($U_{1}$) to a low state and waits for 300 ms. The waiting time was set to be longer than the time constant of the excitation photodiode amplifier ($R_{F2}C_{F2}$ = 22 ms) and the emission photodiode amplifier ($R_{F1}C_{F1}$ = 10 ms) to ensure stable output. The microcontroller reads the outputs of the photodiode amplifier ($V_{A1}$ and $V_{A3}$), as well as the reference voltages ($V_{A2}$ and $V_{A4}$), using the ADC operating in the differential mode. The averaged values of the photodiode amplifier outputs are subtracted from the corresponding reference voltage and output when the LED is off to reduce errors. Fluorescence intensity (F) is calculated using the following formula:

(5) $$F=\left[{\left(V_{A3}-V_{A4}\right)}_{AVG,\;LED\;ON}-{\left(V_{A3}-V_{A4}\right)}_{AVG,\;LED\;OFF}]/[{\left(V_{A1}-V_{A2}\right)}_{AVG,\;LED\;ON}-{\left(V_{A1}-V_{A2}\right)}_{AVG,\;LED\;OFF}\right],$$

where AVG indicates that the quantities are averaged and the other variables are as shown in Figure 5.

#### 2.4.4. Noise and Variation Analysis

Various techniques (Table 1) were employed to analyze and mitigate the noise and variation of the fluorometer, thus ensuring high accuracy. Device-to-device variation occurs as a result of differences between individual devices due to variations in the manufacturing process and component specifications. Run-to-run variation arises over time due to component degradation. Sample-to-sample variation occurs when users change the measuring tube, while in-run variations persist throughout the measurement process. Calibration can help mitigate device-to-device and run-to-run variations caused by manufacturing and component degradation. Changes in the fluorescence chamber design can address sample-to-sample variation resulting from the measuring tube position and sample quantity variation. The measuring tube holder was designed to accommodate measuring tubes with the same conical angles, fitting loosely on the top. This design allows users to drop the measuring tube into the fluorometer without applying pressure, minimizing position variations. Lowering the measurement position of the measuring tube can help mitigate errors caused by sample quantity variation.

## 3. Results

### 3.1. Calibration of Low-Cost, Portable Fluorometer

**Figure 8:**
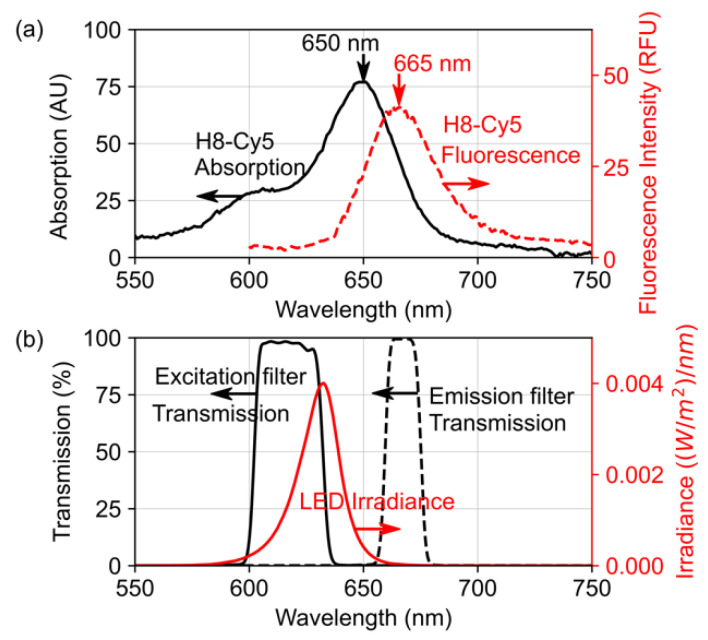
Spectra of the LED, the excitation filter, and the emission filters are matched with the absorption and fluorescence emission spectra of H8-Cy5. (**a**) Measured absorption and fluorescence intensity spectra of H8-Cy5 show central wavelengths at 650 nm and 665 nm, respectively. (**b**) Measured LED irradiance, excitation filter transmission, and emission filter transmission show central wavelengths at 632 nm, 615 nm, and 667 nm, respectively.

The optical components of the fluorometer were carefully chosen to optimize the accuracy of albuminuria quantification. The spectra of the fluorescence-labeled aptamer (H8-Cy5) absorption and fluorescence represent the spectral characteristics of the aptasensor when the aptamer is released from GO and binds to albumin. Figure 8 illustrates the H8-Cy5 spectra, LED irradiance, and filter transmission, while Table 2 provides the central wavelength and FWHM values. The absorption and fluorescence spectra of the Cy5 fluorescence-labeled aptamer (Figure 8a) were measured using a UV–Vis–NIR spectrophotometer (Agilent Technologies Cary 5000) and a fluorescence spectrometer (Perkin Elmer LS55), respectively. Figure 8b displays the LED irradiance and the transmission of the excitation and emission filters. 

To measure the filter transmission and LED irradiance, a spectrometer (Ocean Optics HR4000CG-UV-NIR) with a UV–VIS–NIR light source (Ocean Optics DH-2000-BAL, Ocean Insight, Orlando, FL, USA) was utilized. The LED irradiance was measured at the center of the fluorescence chamber, which corresponded to the location of the measuring tube during the measurement process. The measured spectra indicated that the excitation filter efficiently allows the LED light to excite the aptamer–Cy5 without leaking through the emission filter. Moreover, the emission filter’s transmission band was found to align with the central wavelength of the fluorescence emission spectrum, ensuring the accurate measurement of fluorescence intensity while avoiding autofluorescence from other substances [38].

### 3.2. Performance Comparison of Developed vs. Commercial Fluorometer Using Aptasensor for HSA Detection

#### 3.2.1. Comparison of Calibration Curves for HSA Concentration Measurement: Developed Fluorometer vs. Commercial Fluorometer

**Figure 9:**
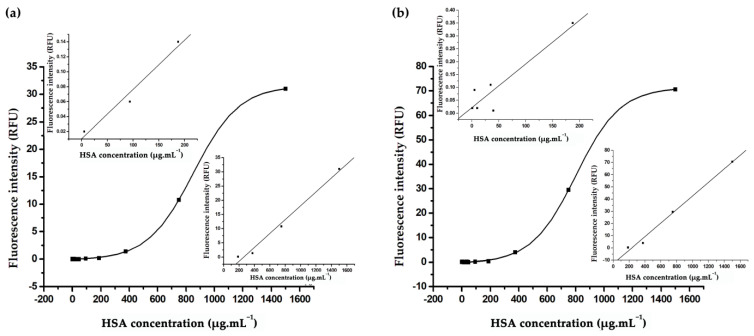
Calibration curves for HSA detection using an aptasensor with two different fluorometers: (**a**) the Quantus^TM^ commercial fluorometer and (**b**) the developed fluorometer. The curves plot fluorescence response against HSA concentration. Both devices exhibited sigmoidal correlation within the 0–1.6 mg/mL range. The Quantus^TM^ commercial fluorometer results show two linear correlations, one between 0 and 200 μg/mL (upper graph (**a**)) with Y = 0.0006593(X) + 0.01042 (R^2^ = 0.98485) and another between 200 and 1600 μg/mL (lower graph (**a**)) with Y = 0.02442(X) − 6.34609 (R^2^ = 0.99374). The developed fluorometer results also display two linear correlations, one within 0–200 μg/mL (upper graph (**b**)) with Y = 0.0017(X) + 0.0213 (R^2^ = 0.93056) and another between 200 and 1600 μg/mL (lower graph (**b**)) with Y = 0.5557(X) − 12.98087 (R^2^ = 0.9961).

To establish a calibration curve for albumin detection, the developed fluorometer was utilized in conjunction with the aptasensor procedure described in Section 2.1, Section 2.2 and Section 2.3. The aptasensor’s GO and aptamer components were combined in a dark environment, with an incubation time of 5 min at room temperature, forming the GO–aptamer complex. Purified HSA solutions with varying concentrations ranging from 0.001 to 1.5 mg/mL were prepared in the optimized PBS and artificial urine system for the aptasensor. The fluorescence intensities obtained with the developed fluorometer were plotted against the corresponding HSA concentrations, as shown in Figure 9. These results were then compared with those obtained using a commercial fluorometer (Quantus^TM^, Promega).

The calibration curve displayed a sigmoidal correlation within the 0–1.6 mg/mL range. Both the Quantus^TM^ commercial fluorometer and the developed fluorometer showed two linear correlations: one between 0 and 200 μg/mL (Quantus^TM^ fluorometer: Y = 0.0006593(X) + 0.01042, R^2^ = 0.98485; developed fluorometer: Y = 0.0017(X) + 0.0213, R^2^ = 0.93056) and another between 200 and 1600 μg/mL (Quantus^TM^ fluorometer: Y = 0.02442(X) − 6.34609, R^2^ = 0.99374; developed fluorometer: Y = 0.5557(X) − 12.98087, R^2^ = 0.9961).

The limits of detection (LODs) were determined to be 40 ng/mL for the Quantus^TM^ fluorometer and 203 ng/mL for the developed fluorometer, which were impressively lower than those of the immunoturbidimetry method by 150-fold and 30-fold, respectively [35], showcasing the excellent sensitivity of both portable fluorometers for albumin detection. The developed fluorometer’s LOD was 5-fold higher than that of the commercial fluorometer due to some environmental light leakage during the onsite field test. This issue can be addressed by improving the fluorometer case design and material to completely block external light. Overall, the sensitivity is more than sufficient for screening and monitoring kidney function by detecting albuminuria.

#### 3.2.2. Comparison of HSA Detection in Urine Samples Using the Developed Fluorometer, Commercial Fluorometer, and Standard Hospital Method

**Figure 10:**
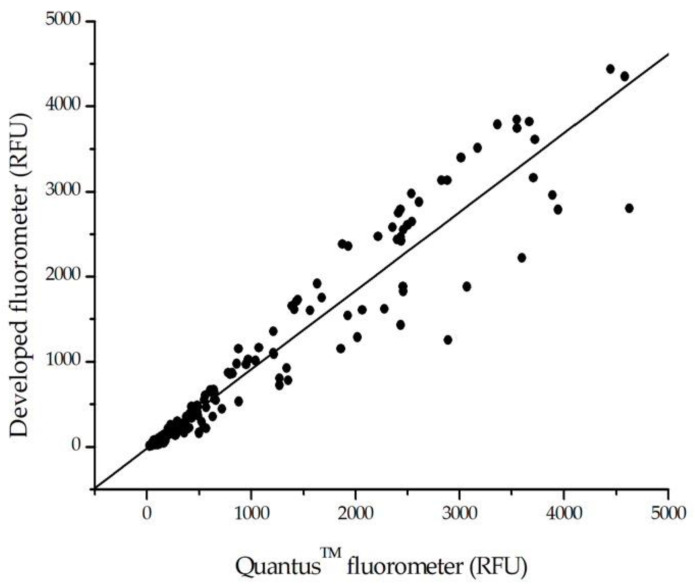
Correlation between the fluorescence intensities measured by the developed fluorometer and the commercial fluorometer in the analysis of albumin concentration in 130 urine samples (black dots). The linear equation for the correlation is y = 0.92628x − 16.134, and the coefficient of determination (R^2^) is 0.9652, indicating a strong positive relationship between the measurements.

The clinical performance of the aptasensor with the developed fluorometer was assessed using 130 urine samples collected from volunteers in the Ubonrat area (Khon Kaen, Thailand). The albumin concentrations in the urine samples were directly measured using the aptasensor and the developed fluorometer without the need for additional treatment. The GO and aptamer were incubated to form the GO–aptamer complex, and the urine samples were added to the complex solution. The fluorescence intensities of the samples were then measured using both the developed fluorometer and a commercial fluorometer. Each sample was tested in parallel using both fluorometers. The albumin concentrations obtained from both fluorometers were analyzed using standard HSA calibration curves (Figure 9), and the corresponding fluorescence intensities are presented in Figure 10. The albumin concentrations determined using the developed fluorometer were comparable to those measured using the commercial fluorometer. The linear equation for the correlation between the fluorescence intensities measured by both fluorometers is y = 0.92628x − 16.134, and the coefficient of determination (R^2^) was calculated as 0.9652, indicating a strong positive relationship between the measurements.

**Figure 11:**
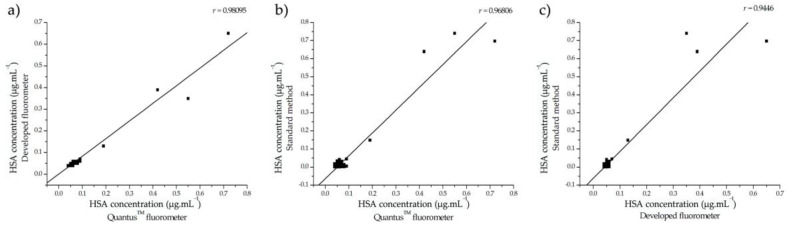
Correlation analysis of measurements obtained using the developed fluorometer, the commercial fluorometer, and the standard method. (**a**) Comparison between the developed fluorometer and the commercial fluorometer, revealing a linear relationship described by the equation y = 0.81496x – 0.00144, with an R^2^ value of 0.98095. (**b**) Relationship between the commercial fluorometer and the standard method, characterized by the equation y = 1.26442x – 0.06416 and an R^2^ value of 0.96806. (**c**) Correlation between the developed fluorometer and the standard method, with a linear equation of y = 1.48508x − 0.06243 and an R^2^ value of 0.9446.

The albumin concentrations measured using the developed fluorometer were compared to the results obtained from the standard method, immunoturbidimetry (Cobas Mira System, Roche Diagnostics, Tokyo, Japan), conducted at Srinagarind Hospital (Khon Kaen, Thailand). The correlation between the albumin concentrations measured by the developed fluorometer and the commercial fluorometer is depicted in Figure 11a. Figure 11b displays the correlation between the commercial fluorometer and the standard method, while Figure 11c illustrates the correlation between the developed fluorometer and the standard method.

These findings revealed a significant correlation between the albumin concentrations determined with both fluorometers and the data obtained through the immunoturbidimetry method, as depicted in Figure 11. Notably, the correlation coefficient between the developed fluorometer and the standard method was 0.9446, indicating a robust correlation. Similarly, the correlation coefficient between the commercial fluorometer and the standard method was 0.96806. These results align closely with those of the standard method and validate the reliability and suitability of the aptasensor integrated with the developed fluorometer for detecting albumin in urine samples without the need for sample dilution.

## 4. Discussion

The aptasensor and portable fluorometer developed in this study exhibited the successful measurement and detection of human serum albumin (HSA) in urine samples. The measured sensitivity limit of detection (LOD) was 0.203 µg/mL, which is comparable to the LOD of the standard method, immunoturbidimetry, at a fraction of the cost. These findings support the feasibility and cost-effectiveness of the aptasensor and fluorometer for HSA detection. 

Although the selectivity of the developed sensor results from the aptamer, which has already been proven to be strongly bound to human serum albumin [28,29], to gain high reproducibility and repeatability from the aptasensor solution, we subtracted the fluorescence signal with the background signal before taking measurements and calculated the percentage fluorescence response instead of the raw fluorescence data. In terms of the fluorometer, the sensor could face long-term optical, mechanical, and electronic component degradation. However, performing calibration before use enhances the reproducibility and repeatability of the fluorometer.

To further the clinical applications of this technology, further investigations are warranted to establish the recommended albumin concentration threshold for the accurate diagnosis of kidney disease. Additionally, it is recommended to explore the utilization of injection-molding techniques for the mass production of the fluorometer’s fluorescence chamber. Rigorous testing, including assessments of shelf life, storage temperature ranges, and drop tests, should be conducted to ensure the reliability of the aptasensor and fluorometer prior to commercialization.

Notably, the user-friendly nature and affordability of the aptasensor, fluorometer, and accompanying software make them suitable for potential commercialization as a home-based kidney disease screening test kit. Such a kit has the potential to facilitate the early screening and monitoring of chronic kidney and renal disease in a convenient and accessible manner.

## 5. Conclusions

In conclusion, this article presents a comprehensive study on the development of an aptasensor coupled with a high-accuracy, low-cost, portable fluorometer and a smartphone application for the measurement of albumin in urine samples, enabling the early detection of kidney disease. The aptasensor incorporates a fluorescence-labeled aptamer in combination with fluorescence-quenching graphene. Remarkably, the aptasensor integrated with the fluorometer achieved an impressive limit of detection (LOD) of as low as 0.203 µg/mL for albumin in urine.

The design of the fluorometer was focused on achieving accuracy while maintaining affordability through the utilization of compact, high-quality optical filters. The fluorometer case was efficiently manufactured using an injection-molding technique, while the circuit board was designed for automatic assembly using pick-and-place machines, facilitating mass production. The portable fluorometer was seamlessly integrated with a smartphone application, enabling the real-time calculation, reporting, and storage of albumin concentration data.

To evaluate its performance, aptasensor-based albumin detection was compared against an immunoturbidimetric assay conducted in a hospital laboratory, utilizing samples from 130 volunteers. The results demonstrated that the aptasensor, fluorometer, and accompanying software exhibit ease of use, a compact size, and low cost, making them highly promising for potential commercialization as home-based albuminuria test kits.

Notably, the portable fluorometer can be easily adjusted for various applications by simply modifying the LED and filters. The software, designed to support multiple users, enables the recording of concentrations for different chemical substances and facilitates long-term health tracking.

Overall, the successful development and validation of the aptasensor, fluorometer, and software highlight their potential as valuable tools for early kidney disease detection, with the added advantages of user-friendliness, affordability, and adaptability. Future efforts should focus on further optimizing and refining these technologies to bring them closer to widespread commercial availability.

## 6. Patents

Some materials in this study have been submitted for intellectual property patents in Thailand (Application number: 19022002705, filing date: 11 July 2019).

## Figures and Tables

**Table 1:** Noise and variation with mitigation techniques.

Time Scale	Noise and Variation	Mitigation Techniques
Device-to-device variation	Manufacturing variation	Factory calibration
Run-to-run variation	Long-term optical, mechanical, and electronic component degradation	Periodic calibration
Sample-to-sample variation	Measuring tube position variation	Measuring tube holder design
	Measuring tube sample quantity variation	Fluorescence chamber design
In-run variation	Johnson–Nyquist noise	Increase averaging time
	Flicker noise	Subtract output measured when LED is on and off
	Temperature fluctuation	Measure both excitation and emission intensity to calculate fluorescence intensity
	External light interference	Opaque case and lid design
	Excitation light leakage	Opaque matte black fluorescence chamber
	Photodiode dark current	Use transimpedance amplifier to read photodiode
	Transimpedance amplifier offset fluctuation	Use operational amplifiers with low input voltage and current offset
	Powerline interference	Decrease bandwidth and increase averaging time

**Table 2:** Central wavelength and FWHM of the optical components and H8-Cy5.

Quantity	Central Wavelength (nm)	FWHM (nm)
Aptamer–Cy5 absorption	649.5	42.7
Aptamer–Cy5 emission	665.0	37.4
LED irradiance	632.3	19.7
Excitation filter transmission	615.0	30.1
Emission filter transmission	667.0	15.8

## Data Availability

Not applicable.
